# Improved Functional Expression of Human Cardiac Kv1.5 Channels and Trafficking-Defective Mutants by Low Temperature Treatment

**DOI:** 10.1371/journal.pone.0092923

**Published:** 2014-03-24

**Authors:** Wei-Guang Ding, Yu Xie, Futoshi Toyoda, Hiroshi Matsuura

**Affiliations:** Department of Physiology, Shiga University of Medical Science, Otsu, Shiga, Japan; Indiana University School of Medicine, United States of America

## Abstract

We herein investigated the effect of low temperature exposure on the expression, degradation, localization and activity of human Kv1.5 (hKv1.5). In hKv1.5-expressing CHO cells, the currents were significantly increased when cultured at a reduced temperature (28°C) compared to those observed at 37°C. Western blot analysis indicated that the protein levels (both immature and mature proteins) of hKv1.5 were significantly elevated under the hypothermic condition. Treatment with a proteasome inhibitor, MG132, significantly increased the immature, but not the mature, hKv1.5 protein at 37°C, however, there were no changes in either the immature or mature hKv1.5 proteins at low temperature following MG132 exposure. These observations suggest that the enhancement of the mature hKv1.5 protein at reduced temperature may not result from the inhibition of proteolysis. Moreover, the hKv1.5 fluorescence signal in the cells increased significantly on the cell surface at 28°C versus those cultured at 37°C. Importantly, the low temperature treatment markedly shifted the subcellular distribution of the mature hKv1.5, which showed considerable overlap with the trans-Golgi component. Experiments using tunicamycin, an inhibitor of N-glycosylation, indicated that the N-glycosylation of hKv1.5 is more effective at 28°C than at 37°C. Finally, the hypothermic treatment also rescued the protein expression and currents of trafficking-defective hKv1.5 mutants. These results indicate that low temperature exposure stabilizes the protein in the cellular organelles or on the plasma membrane, and modulates its maturation and trafficking, thus enhancing the currents of hKv1.5 and its trafficking defect mutants.

## Introduction

Previous studies showed that Kv1.5 underlies the cardiac ultra-rapid delayed rectifier potassium current (*I*_Kur_) in humans [1], [2]. In addition, Kv1.5 channels are also expressed in many other organs, such as pulmonary arteries, brain, skeletal muscle, and have crucial function in cell cycle regulation [3]. Since *I*_Kur_ is expressed in the cardiac atrial, but scarcely in the ventricular cells in human [4], [5], thus *I*_Kur_ has important roles in controlling action potential (AP) shape and contractility in the human atrium. Physiologically, rapid activation of *I*_Kur_ current following the AP upstroke may reduce Ca^2+^ influx via L-type Ca^2+^ channels (*I*_Ca,L_) and result in less positive plateau phase [6]. Whereas, blockade of *I*_Kur_ may facilitate *I*_Ca,L_ activation following a pronounced spike-and-dome configuration and therefore enhance intracellular Ca^2+^ influx [7]. Moreover, human Kv1.5 (hKv1.5) is encoded by KCNA5 and KCNA5 loss-of-function mutations have been reported to produce kindred atrial arrhythmia, which suggest that the genetic alteration of hKv1.5 may substantially enhance arrhythmia susceptibility [8], [9]. Therefore, the normal expression or channel activity of Kv1.5 is important in the regulation of the proper electrical properties of cardiac atrial myocytes. Recently, basic studies have demonstrated that the contribution Kv1.5 can be modified through alterations in the surface density of the channel protein in addition to that of the more classical pore block by drugs [10], [11].

Similar to other ion channels, the Kv1.5 channel protein is assembled at an early stage in channel synthesis in the endoplasmic reticulum and is transferred thereafter through the Golgi apparatus to the plasma membrane [3], [12]. It has been reported that several regulatory proteins and lipids, such as SAP97 [13], half LIM protein 1 [14], β-accessory protein KChIP2 [15], eicosapentaenoic acid [16] and an antiarrhythmic drug, quinidine, [10] regulate Kv1.5 expression and intracellular trafficking processes. On the other hand, in addition to these pharmacological approaches, the sub-physiological culture of mammalian cells (28∼34°C) also resulted in changes in gene expression, culture longevity and recombinant protein production [17]. Regarding ion channels, hypothermic cultivation was firstly reported to rescue the surface expression of trafficking-defective mutants of the cystic fibrosis transmembrane conductance regulator (CFTR), which is a plasma membrane Cl^-^ channel [18]. In addition, low temperature treatments not only markedly rescue the expressions of most trafficking-deficient mutants of Kv11.1 (*human ether-a-go-go related gene*; hERG) channels, but also improve the expression of its wild-type (WT) channels [19]–[21]. However, the detailed mechanisms involved in the actions of the above-reported regulatory proteins and hypothermic treatment remain, for the most part, not clearly understood. In light of these studies, we herein investigated the effects of low temperature exposure on hKv1.5 channel activity, protein expression, maturation, degradation and possible trafficking changes.

Our results provide the first detailed evidence to suggest that reduced-temperature (even at 34°C) culture substantially enhanced the hKv1.5 currents, which is associated with stabilization of the channel protein, slower degradation of its immature protein and the accumulation of mature protein on the plasma membrane.

## Materials and Methods

### Plasmids and Cells

The mammalian expression vector, pcDNA3.1, containing the hKv1.5 cDNA (kindly provided by Dr D Fedida, University of British Columbia, Vancouver, Canada) was used for the expression of all constructs in this study. Stable cell lines constantly expressing hKv1.5 were generated from CHO (CHO-hKv1.5s) and HEK-293 (HEK-hKv1.5s) cells. Polymerase chain reaction (PCR)-based site-directed mutagenesis was applied to introduce mutations into hKv1.5 cDNA using Quikchange Kit (Agilent Technologies, USA, 200521). All PCR-products were fully sequenced (ABI3100x/, Applied Biosystems, USA) to ensure the fidelity of the PCR reactions. The cells were maintained in Dulbecco's modified Eagle's medium/Ham's F-12 (DMEM/F-12, Nacalai, Japan, 08460-95 for CHO cells) or DMEM (Nacalai, 08458-45 for HEK-293 cells) supplemented with 10% fetal bovine serum and antibiotics (100 IU ml^−1^ penicillin and 100 mg ml^−1^ streptomycin) under a humidified atmosphere of 5% CO_2_/95% air at 37°C. The cells were passaged twice weekly by harvesting with trypsin-EDTA, and a part of the treated cells were seeded onto glass coverslips for the patch clamp experiment. In some experiments, the hKv1.5 WT or mutants (I502A and I508A) were transiently transfected into CHO cells together with green fluorescent protein (GFP) cDNA (0.5 μg WT or mutant hKv1.5 +0.5 μg GFP) by using Lipofectamine method according to the manufacture's instructions (Invitrogen, Carlsbad, USA, 18324-012).

### Solutions and reagents

The bath solution (Tyrode's solution) contained (in mM) 140 NaCl, 5.4 KCl, 1.8 CaCl_2_, 0.5 MgCl_2_, 0.33 NaH_2_PO_4_, 5.5 glucose and 5.0 HEPES (pH was adjusted to 7.4 with NaOH). The pipette solution contained 70 mM potassium aspartate, 40 mM KCl, 10 mM KH_2_PO_4_, 1 mM MgSO_4_, 3 mM Na_2_-ATP (Sigma, A7699), 0.1 mM Li_2_-GTP (Roche Diagnostics GmbH, Germany, 10106399001), 5 mM EGTA and 5 mM HEPES (pH was adjusted to 7.2 with KOH). Several reagents, such as Cycloheximide (Sigma, 01810), MG132 (Sigma, C2211), Chloroquine (Sigma, C6628), Tunicamycin (Sigma, T7765) and NH_4_Cl (Sigma, A0171), were used in the present study.

### Electrophysiological recordings and data analysis

The whole-cell membrane currents [22] were recorded with an EPC-8 patch-clamp amplifier (HEKA, Lambrecht, Germany), and the data were low-pass filtered at 1 kHz, acquired at 5 kHz through an LIH-1600 analogue-to-digital converter (HEKA) and stored on a hard disc drive, by using Pulse/Pulse Fit software (HEKA). The patch electrodes had a resistance of 2.5–3.0 MΩ when filled with the pipette solution. The cells attached to the glass coverslips were transferred to a recording chamber (0.5 mL in volume) mounted on the stage of an inverted microscope (ECLIPSE TE2000-U, Nikon, Japan). The chamber was maintained at 25°C and was perfused continuously at a rate of 2 ml min^−1^ with Tyrode solution. In the whole-cell configuration, average series resistance was 5.4±0.1 MΩ. The series resistance was usually compensated by 80%. The hKv1.5 currents were elicited by applying 300-ms depolarizing steps from a holding potential of −80 mV to various levels. The interval between the voltage-clamp steps was 10 s; this allowed the channels to recover fully from possible inactivation between the voltage steps.

### Total membrane extraction

The cells were harvested in a homogenization buffer (50 mM Tris/HCl, 150 mM NaCl, 1 mM EDTA, protease inhibitor cocktail, pH 7.5). The homogenates were centrifuged at 4°C and 50000 rpm for 20 min in a TLA 100.4 rotor by a TLX centrifuge (Beckman), and the pellets were solubilized with 50 μl lysis buffers (10 mM HEPES, 150 mM NaCl, 1 mM EDTA, protease inhibitor cocktail, pH 7.5) and then centrifuged at 100,000 g for 20 min in a TLA 100.4 rotor. The supernatant was used as the total membrane fractions.

### Plasma membrane extraction

The cells were separated into the plasma membrane and cytosolic soluble fractions using a Plasma Membrane Protein Extraction Kit (BioVision, USA, K268-50) according to the manufacturer's instructions.

### Western blot analysis

The protein extractions were quantified by using the Bio-Rad protein dye assay reagent. The lysates containing equal amounts (15 μg per lane) of protein were heated for 3 min at 100°C in Laemmli loading buffer and were fractionated on 5∼20% gradient SDS-polyacrylamide gels (Wako, Japan). The fractionated proteins were thereafter electro-transferred to polyvinylidene difluoride (PVDF) membrane (Bio-Rad, USA). Blue Stat molecular weight markers (Genetics, Japan) were used. The blots were blocked by incubation for 2 h with 5% nonfat milk in Tris-buffered saline (TBS) containing 0.1% Tween 20 (TBST) and were hybridized overnight at 4°C with an anti-hKv1.5 rabbit polyclonal IgG (1∶2000, Chemicon, AB9786), anti-GAPDH rabbit polyclonal IgG (1∶5000, Cell signaling, 0312010), anti-GM130 mouse monoclonal IgG (1∶5000, BD falcon, 610822), anti-TGN46 rabbit polyclonal IgG (1∶5000, Sigma, T7576), anti-Calnexin mouse monoclonal IgG (1∶5000, BD falcon, 610523), and anti-Na^+^/K^+^ ATPase Beta 1 rabbit polyclonal IgG (1∶2000, GeneTex, GTX113390). After washing 3 times for 10 min each with TBST, the blots were incubated for 2 h with horseradish peroxidase-linked anti-rabbit IgG (1∶5000, Amersham, NA934VS) or anti-mouse IgG antibodies (1∶30000, Jackson ImmunoResearch, 115035174). After extensive washing with TBST, the labeling was visualized by chemiluminescence using ECL Western blotting detection reagents. The intensity of the protein bands was quantified using the Image Fuji software (Fuji, Japan) and was normalized to the density of the GADPH positive band.

### Immunofluorescence

The cells were fixed with 4% paraformaldehyde in PBS (pH 7.4) for 20 min and permeabilized with 0.2% TritonX in PBS (PBST) for 10–20 min. To prevent non-specific binding, cells were treated with 10% blocking one (Nacalai, Japan, 03953-95) in PBST for 30 min. The cells were subsequently incubated over night at 4°C with anti-hKv1.5 rabbit polyclonal IgG (1∶2000). The cells were washed thereafter with PBST and incubated for 2 h with Alexa 488-conjugated goat anti-rabbit IgG (1∶500, Molecular Probes, A11008). The cells were rinsed with PBST before their observation under a confocal microscope (LSM META 510). To quantify the effect of low temperature exposure on the intracellular expression of the hKv1.5 protein, we analyzed the fluorescence intensity on the cell membrane [23]. The fluorescence signals of hKv1.5 were measured using ImageJ software.

### Biochemical fractionation

The cells were homogenized in a homogenization buffer (130 mM KCl, 25 mM NaCl, 1 mM EDTA, 25 mM Tris and protease inhibitors, pH 7.4) followed by an additional 12 strokes in 1-ml syringes fitted with a 26-gauge needle. The samples were centrifuged at 600 g for 10 min and the supernatant was used as the post-nucleus supernatant. Thereafter, 900 μl of the post-nucleus supernatant was applied to the top of a 2.5–30% iodixanol discontinuous gradient, consisting of 30, 25, 20, 15, 12.5, 10, 7.5, 5, and 2.5% iodixanol (v/v) in homogenization buffer. The gradient was centrifuged for 1 h at 100000 g in a rotor (Model SW41; Beckman Coulter, Fullerton, CA) at 4°C. Twelve fractions were collected from the top of the tubes by using a plastic pipet. The fraction samples were used directly for Western blotting analyses.

### Statistical analysis

All of the average data are presented as the mean ± SEM. The statistical comparisons between the two groups were analyzed using Student's unpaired *t*-test. The comparisons among multiple groups were evaluated using ANOVA, followed by Dunnett's test, and the differences were considered to be significant at *P*<0.05.

## Results

### Low temperature cultivation modulates the hKv1.5 and its pore mutant currents

**Figure 1:**
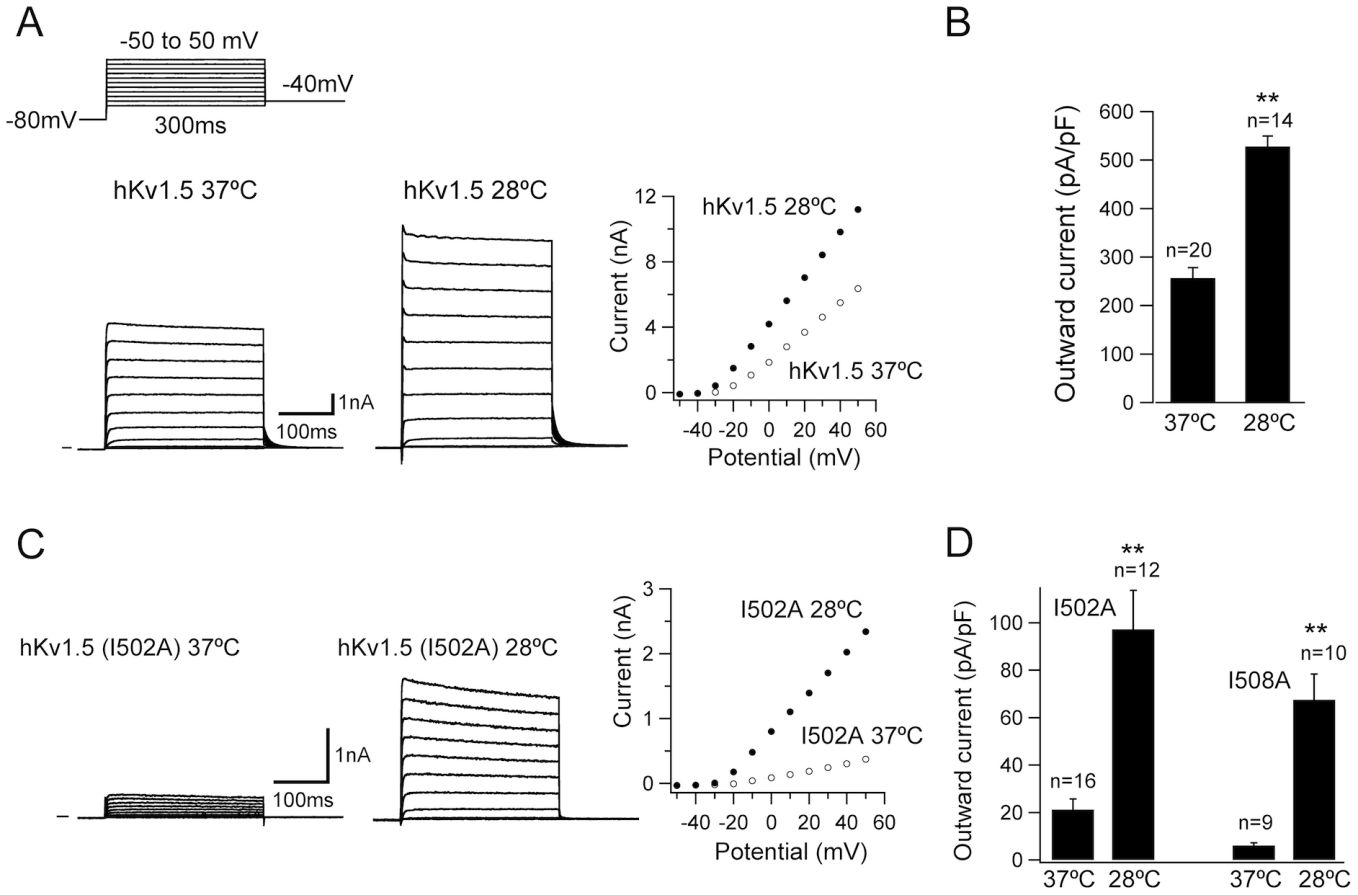
Low temperature cultivation increases the hKv1.5 and its mutant currents. (A) The hKv1.5 currents in CHO-hKv1.5s cells were evoked by 300-ms depolarizing voltage-clamp steps, from a holding potential of −80 mV to various test potentials (−50 to +50 mV), with a return potential of −40 mV, cultured at 37°C (left panel) and 28°C (middle panel) for 48 h. The current-voltage relationship of hKv1.5 currents obtained at different temperature (right panel). (B) Bar graph of the averaged outward current density measured at the end of a 300-ms depolarizing step to +30 mV. (C) The mutant currents recorded by the same protocol from the CHO cells transiently expressing hKv1.5 I502A, cultured at 37°C (left panel) and 28°C (middle panel) for 48 h. (D) Averaged outward current density for the mutant channels (I502A and I508A) measured at the end of a 300-ms depolarizing step to +30 mV (**P<0.01 vs 37°C culture).

As shown in Figure 1, the CHO-hKv1.5s cells were maintained at 28°C for 48 h after pre-culturing at 37°C for 24 h; cultured cells at 37°C for 72 h (24 h+48 h) served as a control. The hKv1.5 currents were evoked by 300-ms depolarizing voltage-clamp steps, from a holding potential of −80 mV to various test potentials (−50 to+50 mV) with a return potential of −40 mV. Under low temperature cultivation (28°C), the hKv1.5 channel activity was significantly elevated in comparison to those cultured at 37°C (Fig. 1A). The current average densities at the +30 mV step approximately doubled from 256.9±12.4 pA/pF to 528.1±21.1 pA/pF (Fig. 1B). The currents recorded were activated rapidly upon depolarization to reach a peak and stabilized thereafter during the moderately depolarized test steps (≤ +30 mV), but decayed minimally during the strongly depolarized test potentials (≥40 mV), which is consistent with the previous studies [24], [25], thus indicating that low temperature cultivation provoked the elevation of the hKv1.5 currents without any characteristic changes. Since reduced temperature culture rescued the currents of trafficking-defective mutants of CFTR [18] and hERG channels [21], we investigate whether low temperature treatment could modify the function of hKv1.5 mutant channels. We and other groups identified several amino acid residues in hKv1.5 (such as I502, I508, located in the pore region), as important sites for drug interactions [24], [26]. Futhermore, when recording from CHO cells transiently expressing hKv1.5 WT or mutants (I502A, I508A), the current amplitude of the mutants was much smaller than that of WT channel, suggesting that these mutations cause a defect in protein transport (487.1±12.4 pA/pF for WT current; 21.4±4.5 pA/pF for I502A current; 6.1±1.1 pA/pF for I508A current, respectively at depolarizing pulse +30 mV). Thus, we tested if low temperature exposure could increase (rescue) the functional expression levels in the mutants. As expected, the current densities measured in the mutants were substantially enhanced at 28°C culture, in comparison to those cultured at 37°C (Fig. 1C and D).

### The hKv1.5 protein levels are altered by low temperature

**Figure 2:**
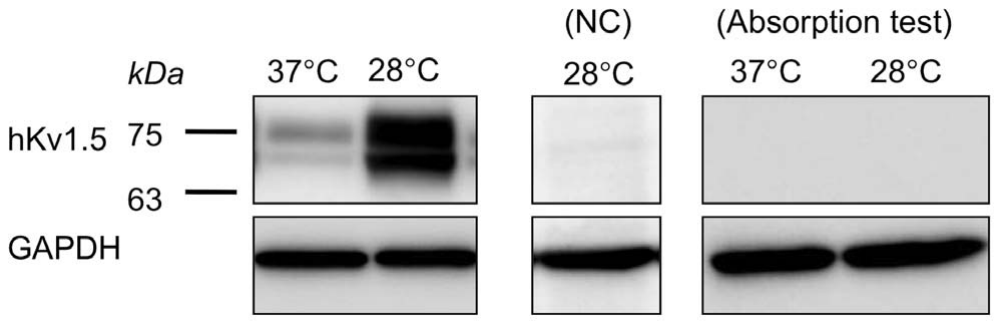
Low temperature exposure increases the protein expression levels of hKv1.5. Western blot analysis of the total membrane proteins extracted from the CHO-hKv1.5s cells (left panel) or native CHO cells (NC, as a negative control) cultured at 37°C or 28°C for 48 h. The specificity of the hKv1.5 antibody was characterized by an antigen absorption test. GADPH was used as control for protein loading.

**Figure 3:**
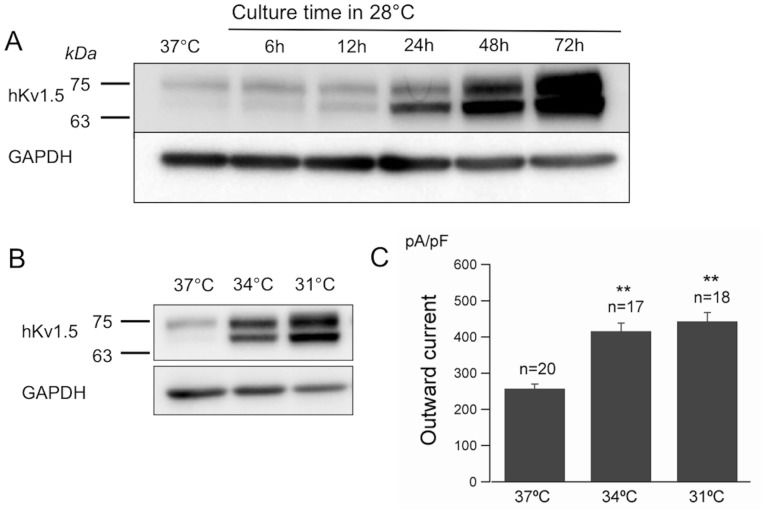
Low temperature cultivation increases hKv1.5 in a time- and temperature-dependent manner. (A) Time chase of the hKv1.5 expression for the 28°C culture at the indicated times. (B) Western blot analysis for the hKv1.5 protein under two conditions of mild hypothermia (31°C and 34°C). (C) A summary of the outward hKv1.5 currents in CHO-hKv1.5s, cultured at 34°C and 31°C (**P<0.01 vs 37°C culture).

To further investigate the phenomenon mentioned above, we determined whether the protein level of hKv1.5 was altered. In the CHO-hKv1.5s cells, Western blot analyses of the total membrane preparations revealed the presence of two immunoreactive bands with a molecular weight of ∼68 and 75 kDa (Fig. 2), due to the different glycosylation status of hKv1.5, as described previously [27]. The low- temperature treatment (28°C) caused a marked increase in the hKv1.5 protein level in comparison to that observed in the control (Fig. 2). However, no positive signals were detected within the native CHO cells (blank control). The specificity of the rabbit polyclonal hKv1.5 antibody was characterized by the antigen absorption test, shown in Figure 2. We thereafter collected the total cell membrane preparations after 6, 12, 24, 48 and 72-h treatments at 28°C for Western blot assays and showed that both the immature (∼68 kDa) and mature (∼75 kDa) hKv1.5 proteins increased in a time-dependent manner (Fig. 3A). Furthermore, we investigated two conditions of mild hypothermia (31°C and 34°C). After a 48-h culture of the cells under these conditions, a Western blotting analysis (Fig. 3B) demonstrated that both of the two mild low temperature cultures also triggered the increase of the hKv1.5 protein, which is associated with the enhanced channel activity measured in the CHO-hKv1.5s cells cultured at such mild hypothermia (Fig. 3C).

### The effects of low temperature on the turnover of hKv1.5

**Figure 4:**
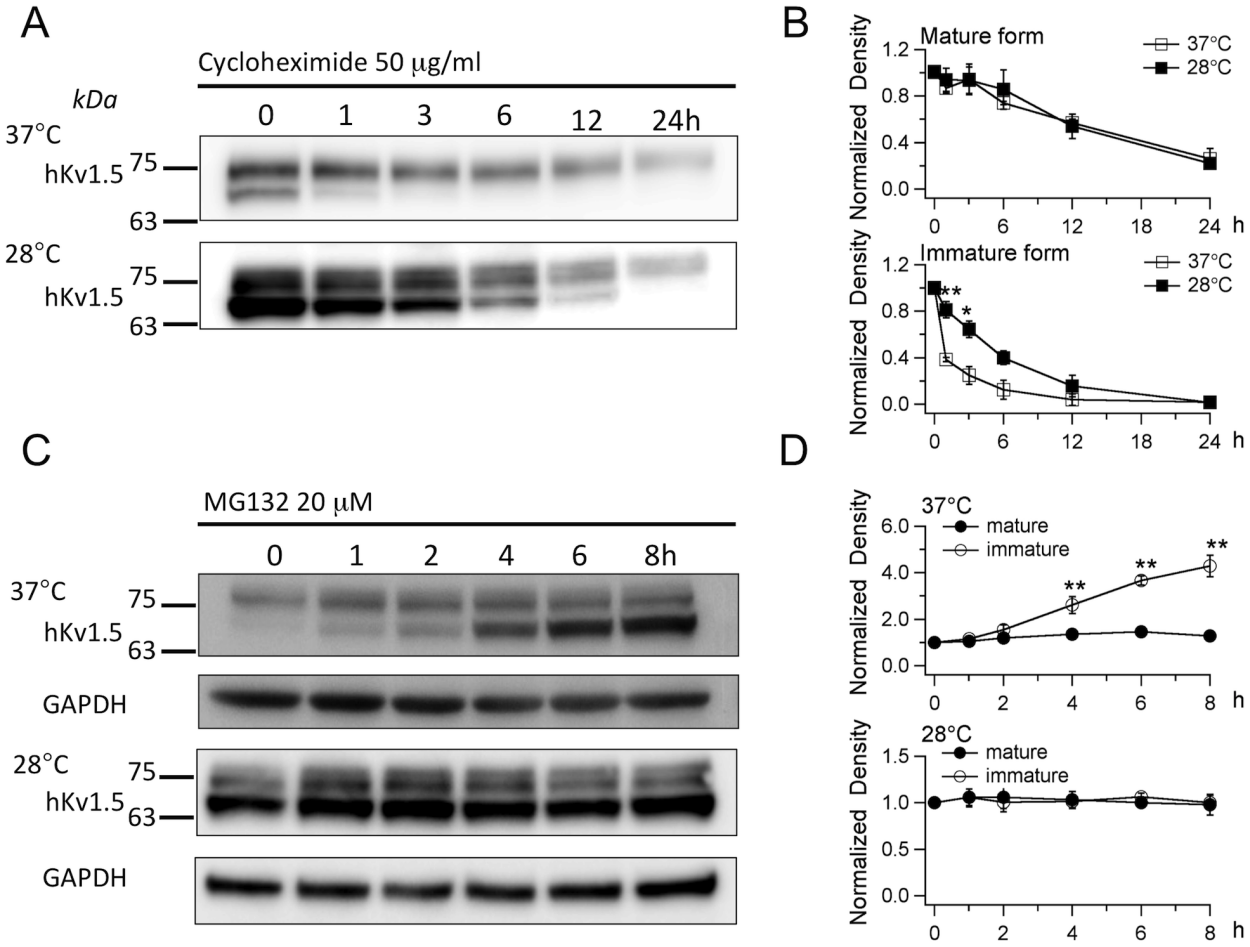
The effects of low temperature culture on the turnover of hKv1.5. (A) Chase analysis of the hKv1.5 expression, cultured at 37°C or 28°C for the indicated times after the treatment with cycloheximide, indicating the prolongation of the decay of the hkv1.5 immature protein cultured at low temperature. (B) Quantitative densitometry scans of the time-chase with cycloheximide treatment, cultured at 37°C or 28°C (*P<0.05 vs 37°C). (C) The expression of hKv1.5 cultured at 37°C or 28°C after the treatment with MG132 (20 μM) for the indicated times. (D) Quantitative densitometry scans of the time-chase with MG132 treatment, cultured at 37°C or 28°C (**P<0.01 vs time 0).

Since the total quantity of the hKv1.5 protein increased due to low temperature cultivation, thus it is logical to assume that the turnover of hKv1.5 was altered under these conditions. Cycloheximide was used to determine the turnover of hKv1.5 by blocking new protein synthesis. The CHO-hKv1.5s cells were first incubated for 48 h at 28°C and 37°C. This was followed by treatment with cycloheximide (50 μg/ml) for 2, 4, 6, 8 and 12 h at 28°C and 37°C, respectively. The results showed that there was obvious variance in the turnover ratios between the 28°C and 37°C cultivations, thus indicating that the turnover of the immature hKv1.5 protein, but not the mature ones, was prolonged under low temperature treatment (Fig 4A and B). Therefore, it is necessary to study whether low temperature cultivation alters the degradation rate of hKv1.5. The protein degradation is generally mediated through two major systems, ie, the proteasome and the lysosome. These different pathways of proteolysis can be differentiated by their sensitivity to proteasome- or lysosome-specific inhibitors. Proteasomal proteolysis can be inhibited by MG132, ALLN and by the fungal metabolite, lactacystin [28]–[30]. The degradation of proteins by the lysosome can be inhibited with chloroquine and ammonium chloride [31].

For this purpose, the CHO-hKv1.5s cells were cultured at 28°C or 37°C for 48 h. This was followed by the treatment with lysosomotropic inhibitor chloroquine (50 μM) for 1, 2, 4, 6 and 8 h, respectively. However, chloroquine did not alter the protein levels of hKv1.5 in both the 28°C and 37°C treatments (Figure S1). The same result was also observed with ammonium chloride (20 mM) (Figure S2). Therefore, we excluded the contribution of the lysosomal system in the degradation of hKv1.5 in the CHO cells under the present experimental conditions. Proteasomal proteolysis was thereafter investigated by the application of the proteasome-specific inhibitor, MG132. Similarly, the CHO-hKv1.5s cells were cultured at 28°C or 37°C for 48 h, and then followed by the treatment with MG132 (20 μM) for 1, 2, 4, 6 and 8 h, respectively. Under 37°C cultivation, the treatment with MG132 caused a significant increase in immature hKv1.5 in a time-dependent manner, whereas there was barely any change in the mature hKv1.5 protein (Fig. 4C and D), thus suggesting that the proteasome partially contributes to the degradation of the immature hKv1.5 but not the mature protein. This result is supported by our electrophysiological data, which show that the MG132 application failed to enhance the currents in the CHO-hKv1.5s cells, cultured at 37°C (256.9±12.4 pA/pF for control; 273.6±41.3 pA/pF after MG132 treatment for 8∼12 h). Furthermore, treatment with MG132 in the CHO-hKv1.5s cells cultured at 28°C for up to 8 h did not cause any alteration of either the mature or the immature hKv1.5 (Fig. 4C and D). It was clearly demonstrated that under both the 28°C and 37°C cultivations, the mature hKv1.5 protein levels were not altered by the MG132 treatments, thus indicating that the abundance of the mature hKv1.5 under reduced-temperature cultivation is not a result of the inhibition of proteolysis.

### Plasma membrane protein sorting of hKv1.5 is promoted by low temperature cultivation

**Figure 5:**
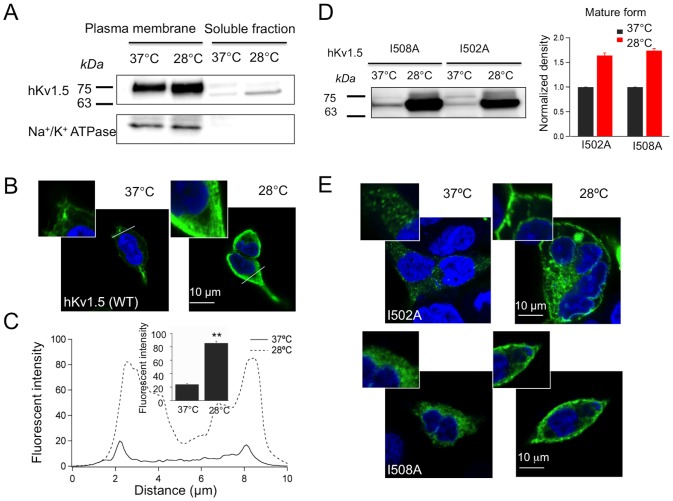
The accumulation of hKv1.5 in the plasma membrane is promoted by low- temperature cultivation. (A) Western blot analysis of the plasma membrane and soluble fractions of the hKv1.5 protein isolated from the HEK-hKv1.5s cells cultured at 37°C or 28°C; Na^+^/K^+^ ATPase beta-1 was used as a plasma membrane marker. (B) Confocal images of the HEK-hKv1.5s cells cultured at 37°C or 28°C. (C) A representative linear plot analysis of the hKv1.5 fluorescent intensity. The x-axis indicates the length across the cell area and the y-axis indicates the fluorescence intensity. The summarized fluorescence intensities measured within the peak of the fluorescent intensity (inset). (D) Western blot analysis for the hKv1.5 mutants (I508A, I502A), transiently expressed in CHO cells, under two temperature conditions (37°C and 28°C). (E) Confocal images of the HEK cells transiently expressing hKv1.5 I502A and I508A, cultured at 37°C or 28°C (**P<0.01 vs 37°C culture).

The degradation of the mature hKv1.5 was not altered by the inhibition of protein hydrolysis in the low temperature culture; therefore, one possible mechanism for hKv1.5 protein increment is through the enhancement of protein maturation. The plasma membrane fractions were isolated from the HEK-hKv1.5s cells cultured at 28°C or 37°C for 48 h, respectively. A Western blotting analysis showed that the cell surface protein level of hKv1.5 increased due to low temperature exposure (Fig. 5A). This strongly suggests that the hypothermic exposure-induced increase of the hKv1.5 currents is partially due to the accumulation of its mature protein, especially on the plasma membrane. Moreover, we employed immunocytochemistry to investigate whether there are changes in the subcellular distribution of hKv1.5. As shown in Figure 5B, the hKv1.5 fluorescence signal increased on the cell surface in the 28°C cultivation in comparison to the control. The quantification of the hKv1.5 fluorescence intensity on the plasma membrane indicated that the low temperature exposure caused an approximately 3-fold increment in the hKv1.5 fluorescence versus the control group (Fig. 5C). Similarly, we found that low temperature exposure at 28°C could also produce a significant increase in protein expression in the mutants I502A and I508A (Fig. 5D). Especially, the mature protein (75-kDa protein band) was minimal in control condition, but was markedly detected in the two mutants incubated at 28°C. In addition, as shown in Figure 5E, the mutant proteins were scarcely transported to the cell membrane, but mostly localized in the cytosol at 37°C culture. In contrast, the cells cultured at 28°C, exhibit greater staining of both mutants on the cell surface.

**Figure 6:**
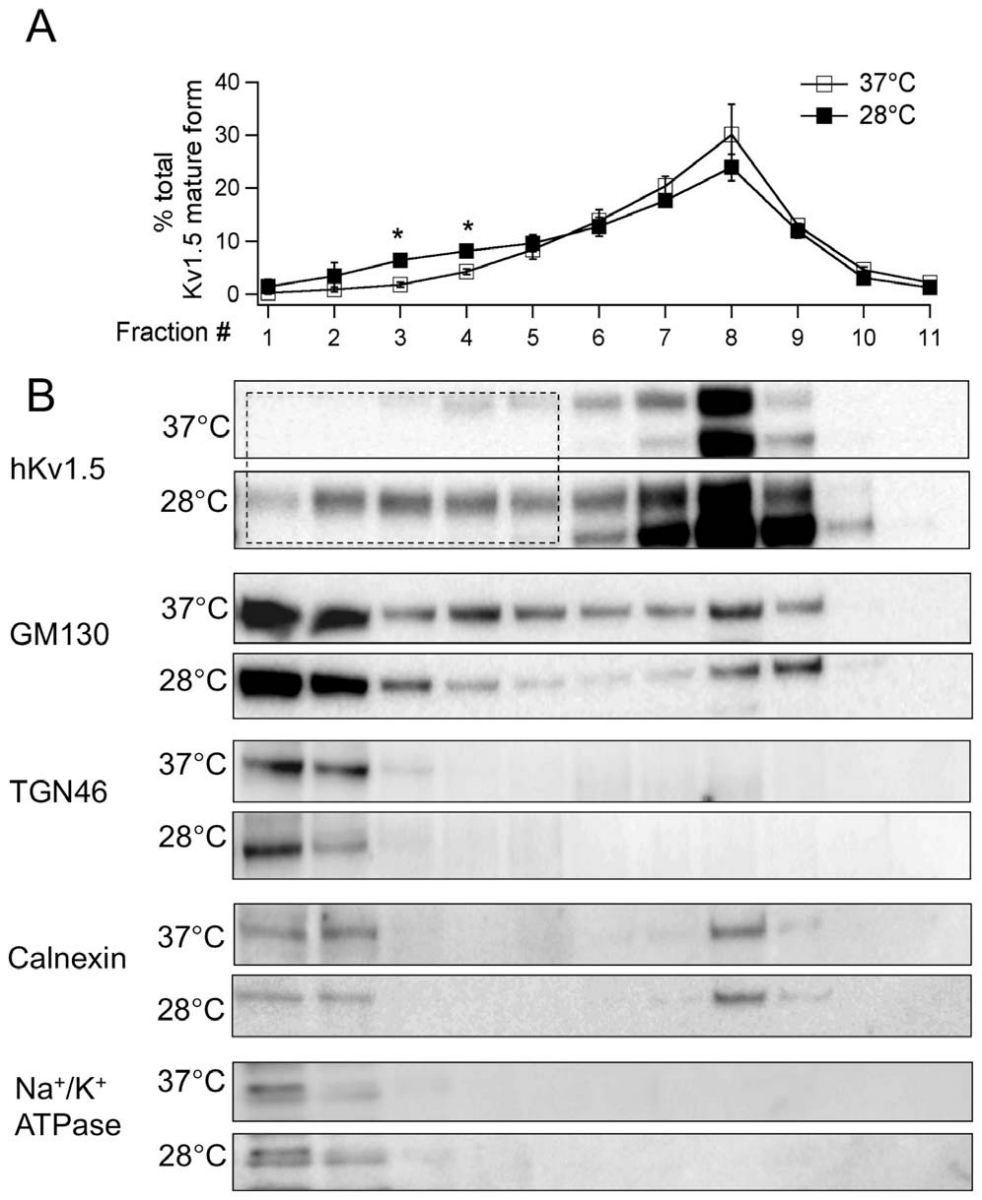
Subcellular fractionations of hKv1.5 and the related marker proteins isolated from the HEK-hKv1.5s cells. The gradient fractionation of extracts, from the HEK-hKv1.5s cells cultured at 37°C or 28°C, was fractionated through iodixanol gradient centrifugation. (A) Densitometry from western blots of hKv1.5 mature form as in B, to allow the distribution of hKv1.5 mature protein to be compared in different temperature (*P<0.05 vs 37°C culture). (B) Western blot analysis showing the subcellular distribution of hKv1.5, GM130 (Cis-Golgi marker), TGN46 (trans-Golgi marker), Calnexin (ER marker) and Na^+^/K^+^ ATPase beta-1 (membrane marker). The dotted line square indicates that the subcellular distribution of the mature hKv1.5 is shifted to the lighter fractions.

To further investigate the mechanism associated with the specific increase of the hKv1.5 protein on the plasma membrane under hypothermic condition, the cells were fractionated into organelles by Iodixanol gradient centrifugation [16], [32]. It is worth noting that the low temperature culture promoted alterations of the hKv1.5's subcellular distribution (Fig. 6). Namely, the subcellular distribution of the mature hKv1.5 was markedly shifted to the lighter fractions, which showed considerable overlap with TGN46, a trans-Golgi network marker, and ATPase beta1, a protein marker of the plasma membrane (Fig. 6A and B). However, the immature hKv1.5 distribution was not altered. Meanwhile, low temperature cultivation did not disrupt the organelles' marker distribution patterns (Fig. 6B), thus indicating that the effect caused by reduced temperature on the hKv1.5 distribution is specific. These results support a view that the low temperature-induced accumulation of the hKv1.5 protein on the plasma membrane and alteration of its subcellular distribution are due in part to the modulation of the maturation and intracellular trafficking processes.

### Expression of N-glycosylated form of hKv1.5

**Figure 7:**
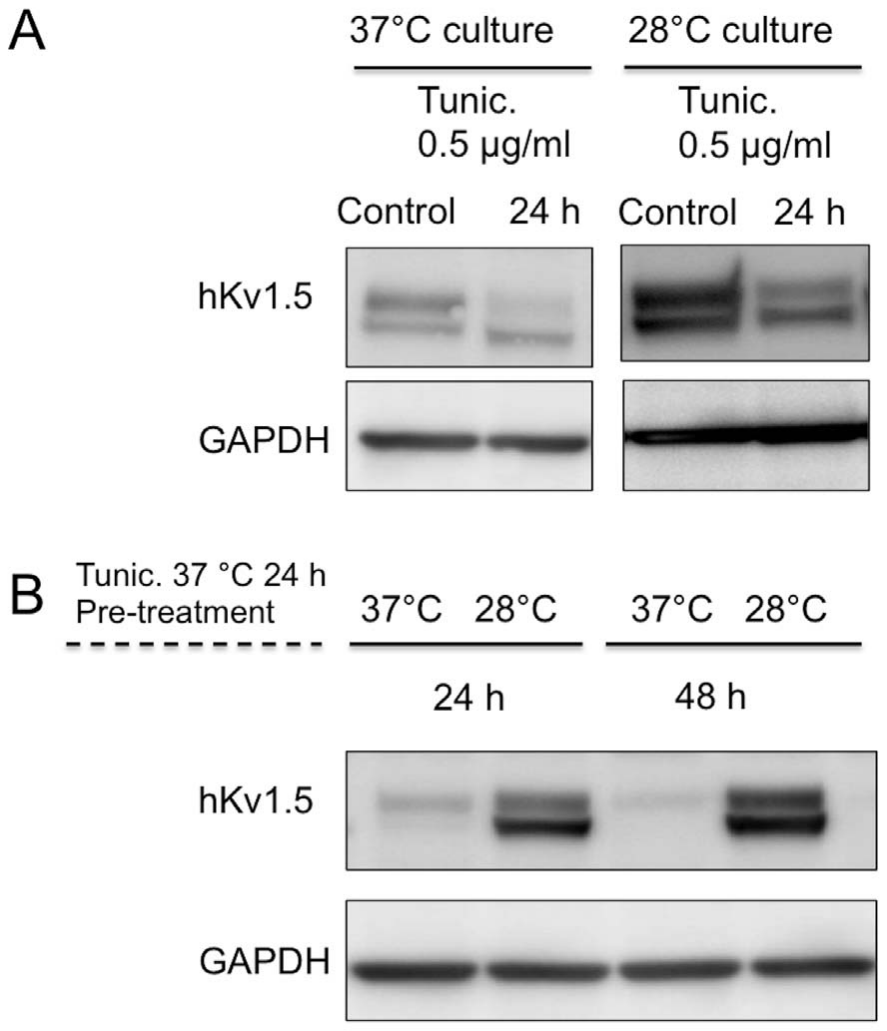
The process of N-glycosylation of hKv1.5 is more effective at 28°C than at 37°C. (A) Western blot analysis of the hKv1.5 expression cultured at 37°C or 28°C for 24 h in the presence of tunicamycin (Tunic), a N-glycosylation inhibitor. (B) The CHO-hKv1.5s cells were pre-treated with tunicamycin for 24 h at 37°C, and then washed to stop its blocking action. The cells were continuously cultured at 37°C or 28°C for the indicated times.

Previous reports have shown that N-glycosylation of many ion channels and membrane receptors not only affects their folding and trafficking but also their gating properties [33], [34]. To address the question whether the N-glycosylation process in hKv1.5 is altered at low temperature, we employed tunicamycin, an antibiotic that blocks the addition of carbohydrate molecules to asparagine residues of related glycoproteins [35]. In the control experiments, after treatment with tunicamycin (5 μg/ml) at 37°C or 28°C [34], there was a marked decrease of the higher molecular weight band (Fig. 7A). Thus, in next experiment, we pre-treated cells with tunicamycin at 37°C for 24 h, washed to remove the drug and then subsequently cultured cells at 37°C or 28°C for 24 and 48 h. Both mature and immature forms of the channel protein were markedly enhanced (Fig. 7B) by low temperature exposure in comparison to those observed at 37°C, thus indicating that the N-glycosylation process of hKv1.5 is effectively performed even at reduced temperature.

## Discussion

In recent years, there have been a growing number of reports on the use of reduced temperature cultivation of mammalian cells for the enhanced production of recombinant protein production and improved product quality, including cardiac channel proteins, such as the hERG channel protein [19]–[21]. The mutant N470D of hERG is trafficking-deficient in cells cultured at physiological temperature, whereas the channel protein transports to the plasma membrane to produce functional channels at reduced temperature [21]. This result is confirmed by later studies showing that most of the mutations of hERG with the trafficking-deficient phenotype are corrected to increase functional expression on the cell surface membrane by low temperature culture [19]. Similarly, reduced-temperature cultivation also improves the expression of the trafficking-defective CFTR mutants in transfected polarized CFBE41o-cells [36]. In addition, some drugs such as thapsigargin and hERG channel inhibitor E-4031 are also reported to promote the functional expression of trafficking-deficient hERG channels [19], thus suggesting that the common mechanism of rescue effects by low temperature may be utilized in the future to understand the regulatory pathways by which the clinical drugs modulate the trafficking process of the ion channels.

Our study demonstrates for the first time that low temperature cultivation (34°C, 31°C and 28°C) markedly increased the hKv1.5 currents. Moreover, we found that hypothermic exposure could also cause an increase of both the immature and mature hKv1.5 proteins in the cell membrane fractions in a time-dependent manner, thus indicating that enhancement of the hKv1.5 channel activity by low temperature exposure might result from alteration of the amount of hKv1.5 protein. Furthermore, our data showed that low temperature could modify the turnover of the immature hKv1.5 protein. However, it is difficult to observe differences for the mature hKv1.5 between temperature treatments because of the long half-life of the mature hKv1.5. We found that the immature hKv1.5 was degraded in the proteasome pathway (MG132-sensitive), but not the mature ones under both experimental conditions, physiological and low temperature exposure. This finding is similar to previous reports showing that the inhibition of the proteasome does not result in an increase of the mature form of CFTR [37] or hERG [38].

The low temperature treatment caused increment of both the immature and mature hKv1.5 proteins without alteration of the degradation of the mature form through the proteasome pathway, thus suggesting that degradation of the immature protein may be prolonged following enhanced glycosylation. It has been revealed that N-glycosylation, a common posttranslational modification of proteins may affect protein folding, maturation, trafficking, stability and consequently expression of functional ion channels [33], [34], [39]. At the endoplasmic reticulum (ER), the addition of asparagine-linked oligosaccharides (core glycosylation) plays a critical role in the quality control of various membrane proteins. Properly folded proteins are then transported to the Golgi apparatus where the carbohydrate chains acquire further maturation prior to expression at the cell surface. The voltage-gated K^+^ channels Kv1.1-Kv1.5 members possess one N-glycosylation consensus site on the extracellular S1–S2 linker [40]. It was reported that N-glycosylation of Kv1.1 channels affects the gating function by a combined surface potential and cooperative channel subunit interaction mechanism [41]. In the present study, we found that the intensity of the higher molecular weight band (mature protein) is markedly decreased by tunicamycin treatment (a N-glycosylation inhibitor), whenever the cells were cultured at 37°C or 28°C for 24 h. This observation suggests that hKv1.5 is a N-glycosylated protein and that tunicamycin is still a potent blocker of N-glycosylation at low temperature. When we conducted an experiment using tunicamycin pre-treatment to allow the cells to start N-glycosylation at the same level, the mature form of hKv1.5 protein is markedly enhanced by low temperature exposure, suggesting that the N-glycosylation process of hKv1.5 is indeed more efficient during low temperature culture.

The surface density of the ion channels results from the balance between the anterograde and retrograde trafficking of these channels [42]. By applying biochemical methods, we found a remarkable increase in the mature hKv1.5 protein in the plasma membrane fraction. Furthermore, we found a marked shift in the subcellular distribution of the mature form to the lighter fractions under low temperature exposure without altering the distribution of the immature forms. This effect is specific to the membrane proteins because reduced-temperature cultivation did not notably alter the intracellular organelles' distributions. Therefore, the sorting of hKv1.5 to the plasma membrane may be enhanced by low temperature through the modification of the maturation and trafficking pathways. From the immunocytochemical observation made in the mutants I502A and I508A, we consider that hypothermic exposure redistributed mutant proteins from predominantly perinuclear intracellular compartment(s) to the cell surface. Thus, the present study suggests that low temperature exposure may provide more time for correct folding and allow these proteins to escape the quality control system and be transported through the Golgi complex to the plasma membrane. However, it should be noted that the exposure to reduced temperature might elicit changes in other cellular responses, such as the cell cycle, metabolism, and cytoskeleton arrangement [17]. Therefore, further studies are needed to clarify whether these mechanisms could also contribute to the low temperature-induced increment of the hKv1.5 currents.

In conclusion, the present study demonstrates that culturing transfected cells at low temperature increases both the channel activity and the protein level of hKv1.5 *in vitro*. This phenomenon may be due in part to the stabilization of the immature hKv1.5 protein and the enhanced intracellular trafficking of the mature protein. The present results may provide insight into the potential mechanisms of different drugs (anti-arrhythmic drugs), which may modify the maturation and trafficking of hKv1.5 channels.

## Supporting Information

Figure S1 **We analyzed the role of lysosomal degradation for hKv1.5 life cycle with lysosomotropic inhibitor, chloroquine under tow temperature conditions.** CHO cells stably expressing hKv1.5 were first cultured for 48 h at 37°C or 28°C. This was followed by treatment with chloroquine (50 μM) for 1, 2, 4, 6 and 8 h at 37°C or 28°C, respectively. The results show that chloroquine treatment did not alter the expression level of hKv1.5, neither the mature nor immature proteins at both cultured temperatures, thus suggesting that the lysosome pathway is not involved in intracellular degradation of the hKv1.5 protein under present experimental condition (n = 4).
(TIFF)

Figure S2 **We analyzed the role of lysosomal degradation for hKv1.5 life cycle with another lysosomotropic inhibitor, ammonium chloride under tow temperature conditions.** CHO cells stably expressing hKv1.5 were first cultured for 48 h at 37°C or 28°C. This was followed by treatment with ammonium chloride (20 mM) for 1, 2, 4 and 8 h at 37°C or 28°C, respectively. The results also show that this treatment did not alter the expression level of hKv1.5, neither the mature nor immature proteins at both cultured temperatures, thus suggesting that the lysosome pathway is not involved in intracellular degradation of the hKv1.5 protein under present experimental condition (n = 3).
(TIFF)
