# Supplementary figures and images for: Improved Functional Expression of Human Cardiac Kv1.5 Channels and Trafficking-Defective Mutants by Low Temperature Treatment
Source: PLoS One. 2014 Mar 24;9(3):e92923. doi: 10.1371/journal.pone.0092923 (PMC3963980; doi:10.1371/journal.pone.0092923)

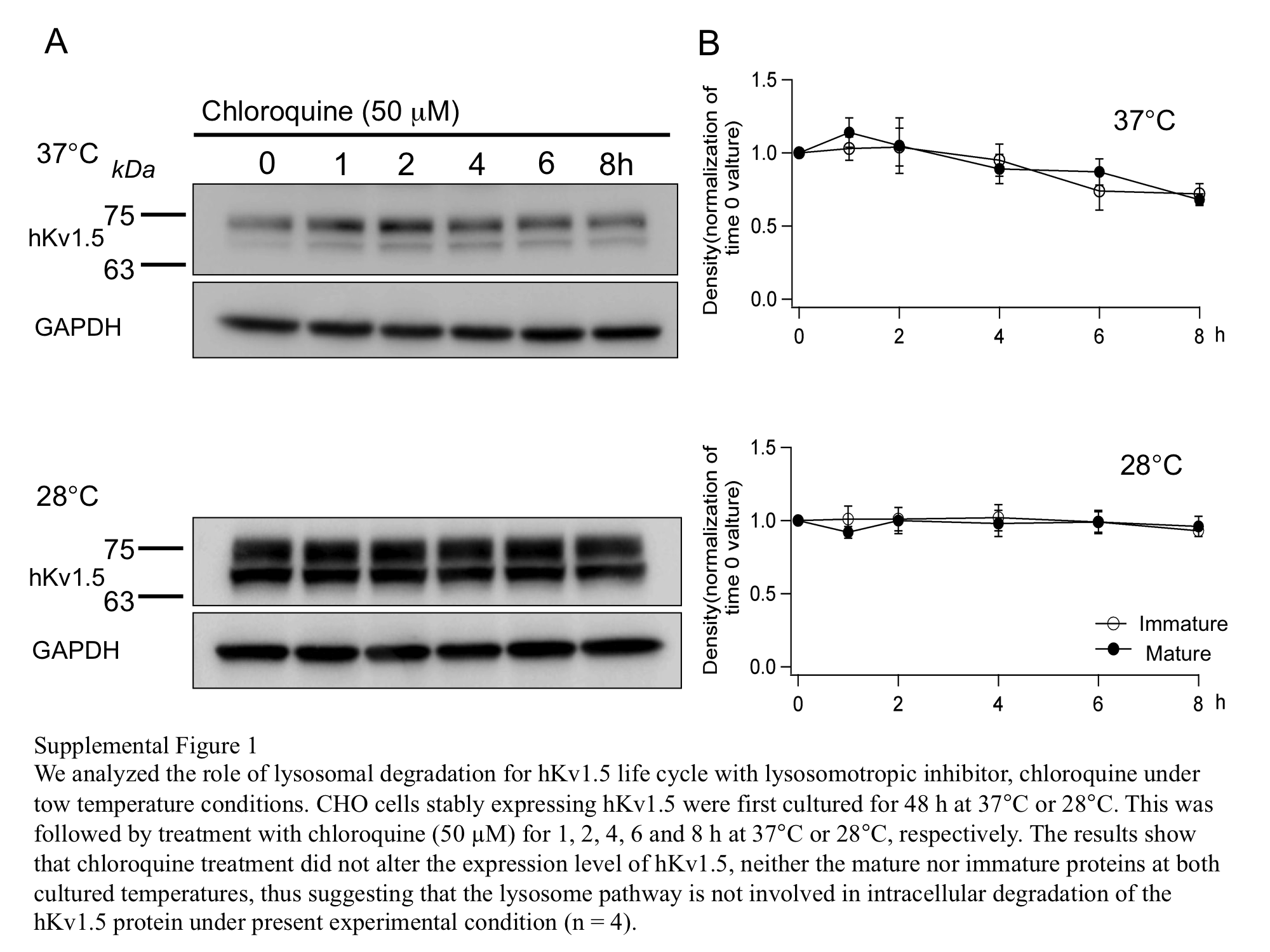

Supplement: Figure S1 — We analyzed the role of lysosomal degradation for hKv1.5 life cycle with lysosomotropic inhibitor, chloroquine under tow temperature conditions. CHO cells stably expressing hKv1.5 were first cultured for 48 h at 37°C or 28°C. This was followed by treatment with chloroquine (50 μM) for 1, 2, 4, 6 and 8 h at 37°C or 28°C, respectively. The results show that chloroquine treatment did not alter the expression level of hKv1.5, neither the mature nor immature proteins at both cultured temperatures, thus suggesting that the lysosome pathway is not involved in intracellular degradation of the hKv1.5 protein under present experimental condition (n = 4). (TIFF) [file pone.0092923.s001.tiff]

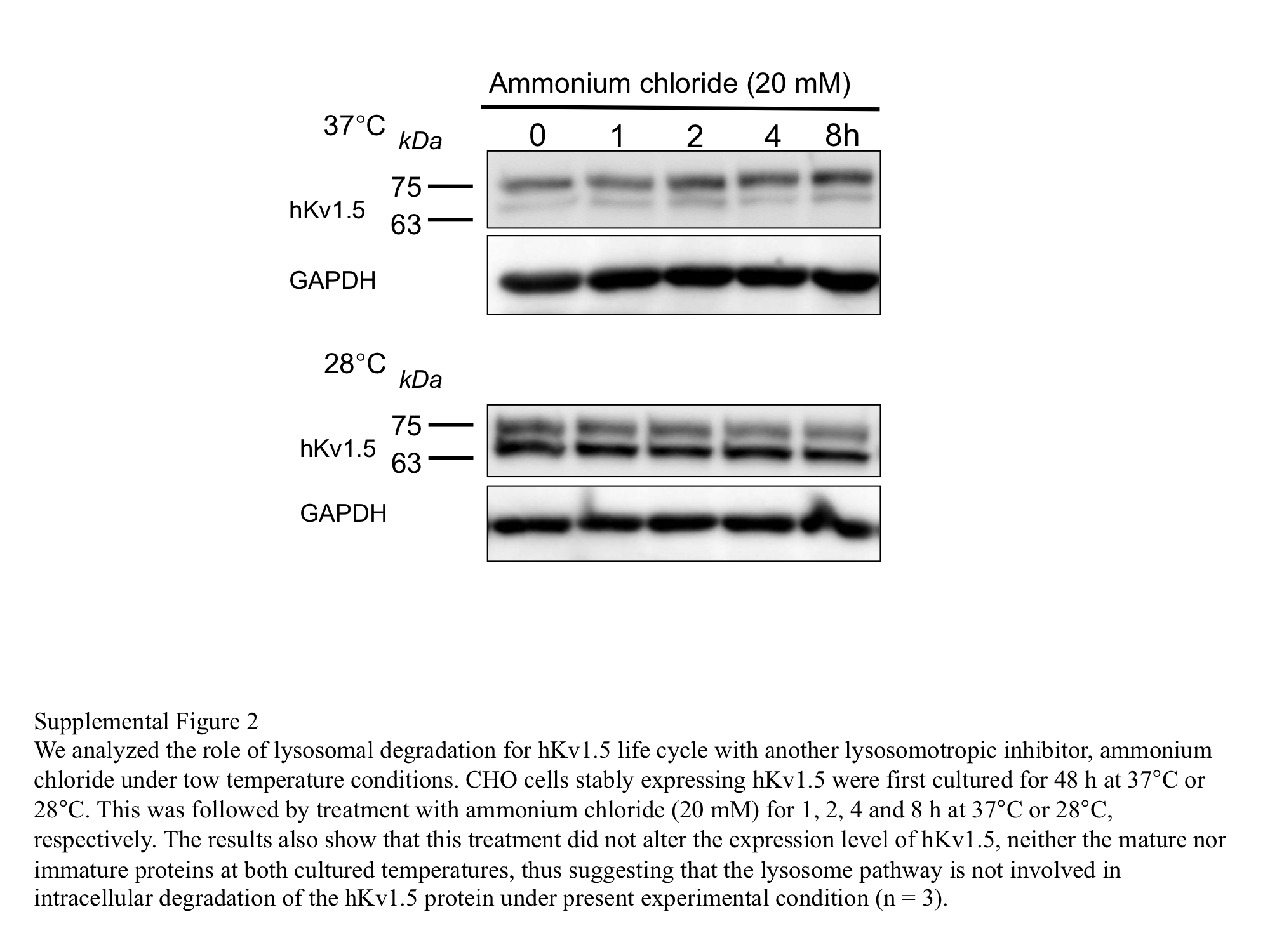

Supplement: Figure S2 — We analyzed the role of lysosomal degradation for hKv1.5 life cycle with another lysosomotropic inhibitor, ammonium chloride under tow temperature conditions. CHO cells stably expressing hKv1.5 were first cultured for 48 h at 37°C or 28°C. This was followed by treatment with ammonium chloride (20 mM) for 1, 2, 4 and 8 h at 37°C or 28°C, respectively. The results also show that this treatment did not alter the expression level of hKv1.5, neither the mature nor immature proteins at both cultured temperatures, thus suggesting that the lysosome pathway is not involved in intracellular degradation of the hKv1.5 protein under present experimental condition (n = 3). (TIFF) [file pone.0092923.s002.tiff]
